# Targeted Inhibition of FAK, PYK2 and BCL-XL Synergistically Enhances Apoptosis in Ovarian Clear Cell Carcinoma Cell Lines

**DOI:** 10.1371/journal.pone.0088587

**Published:** 2014-02-11

**Authors:** Heejei Yoon, Yoon-La Choi, Ji-Young Song, Ingu Do, So Young Kang, Young-Hyeh Ko, Sangyong Song, Byoung-Gie Kim

**Affiliations:** 1 Institute for Refractory Cancer Research, Samsung Medical Center, Sungkyunkwan University School of Medicine, Seoul, Korea; 2 Department of Pathology, Samsung Medical Center, Sungkyunkwan University School of Medicine, Seoul, Korea; 3 Samsung Cancer Research Institute. Samsung Medical Center, Sungkyunkwan University School of Medicine, Seoul, Korea; 4 Department of Obstetrics and Gynecology, Samsung Medical Center, Sungkyunkwan University School of Medicine, Seoul, Korea; The University of Hong Kong, Queen Mary Hospital, Hong Kong

## Abstract

Ovarian clear cell carcinoma (OCCC) displays a higher resistance to first line chemotherapy, requiring the development of new therapeutics. We previously identified a frequent chromosomal gain at 8q24 that harbors the focal-adhesion kinase (FAK) gene; the potential of this gene as a therapeutic target remains to be evaluated in OCCCs. We first examined the dependence of OCCCs on FAK and the PI3K/AKT signaling pathway. FAK was overexpressed in 20% of 67 OCCC samples, and this overexpression was correlated with its copy number gain. *FAK* copy number gains and mutations in *PIK3CA* accounted for about 40% of OCCC samples, suggesting that the FAK/PI3K/AKT axis is an attractive candidate for targeted therapeutics. We, therefore, treated ovarian cancer cell lines, including OCCC subtypes, with the FAK inhibitors PF-562,271 (PF271), and PF-573,228 (PF228). Ovarian cancer cells were more sensitive to PF271 than PF228. We then searched for single agents that exhibited a synergistic effect on cell death in combination with PF271. We found that co-treatment of PF271 with ABT-737, a BCL-2/BCL-XL antagonist, was profoundly effective at inducing apoptosis. RMGI and OVISE cells were more sensitive to ABT-737 than OVMANA and SKOV3 cells, which have *PIK3CA* mutations. Mechanistically, PF271 treatment resulted in the transient down-regulation of the anti-apoptotic protein MCL1 via the PI3K/AKT pathway. Therefore, PF271/ABT-737 treatment led to the inhibition of the anti-apoptotic proteins MCL1 and BCL-XL/BCL-2. We suggest that pharmacological inhibition of BCL-XL and FAK/PYK2 can be a potential therapeutic strategy for the treatment of OCCC.

## Introduction

Ovarian clear cell carcinoma (OCCC) is a subtype of epithelial ovarian cancer and accounts for 5–25% of ovarian cancers [1]. OCCC has shown a high rate of resistance to first-line platinum- and taxane-based chemotherapies, resulting in a worse prognosis [1]. Most common somatic mutations occur in the *ARID1A* (46%), *PIK3CA* (33%), and *PPP2R1A* genes [2], [3]. We previously identified frequent chromosomal copy number gains on 8q24.3, which includes the gene for focal-adhesion kinase (FAK) in OCCC [4]. FAK is a non-receptor tyrosine kinase that localizes to the sites of integrin adhesion and mediates growth-factor signaling, cell proliferation, cell survival, and cell migration [5]. FAK overexpression was associated with poor prognosis and drug resistance [5]–[7]. However, whether genomic alteration underlies FAK overexpression and its potential as therapeutic target remains to be determined in OCCC.

FAK overexpression and phosphorylation confers matrix-independent survival, thus overcoming the apoptotic cell death termed anoikis [5]. Activated FAK enables the recruitment of other scaffold and signaling molecules such as SRC and p85 subunit of the phosphoinositide 3-kinase (PI3K) to focal adhesion sites, consequently activating downstream cell survival signaling, such as through the PI3K/AKT route [8] and the extracellular signal-related kinase (ERK) route [9]. Integrin/FAK/SRC signaling can stimulate the PI3K/AKT and MEK/ERK pathways, either individually or in combination, depending on the cell type [10].

FAK inhibition alone may not be sufficient for effective targeted therapy. Apoptotic cell death was substantially increased by the inhibition of both FAK and EGFR [11], FAK and c-MET [12], or FAK and SRC [13] that function in parallel or in the same signaling pathway. Moreover, cancer cells individually resistant to chemotherapeutic agents or FAK inhibition were sensitive to the combined action of FAK inhibition and these agents [6], [14], [15]. These findings implied that inhibition of compensatory genes or pathways may enhance the efficacy of triggering apoptosis through FAK inhibition. A potential compensatory gene for FAK is the proline-rich tyrosine kinase (PYK2) that has very high similarity with FAK in terms of protein structure, sequence homology, phosphorylation site, activation by integrins, and association with other focal adhesion proteins [16]. Blockade of anticipated redundant genes or signaling pathways might be necessary for effective targeted therapy.

Apoptosis regulation is based on the balance between pro- and anti-apoptotic proteins within cells. Ovarian carcinomas often overexpress anti-apoptotic proteins such as BCL-XL [17] and MCL1 [18] and thereby shift the balance toward survival, which provides them with a major advantage in protecting themselves from chemotherapeutic agents [17], [19], [20]. Down-regulation of either BCL-XL or MCL1 often causes the overexpression of either one not targeted and thus renders cells resistant to induce apoptosis [20], [21]. Therefore, the concomitant down-regulation of these proteins is necessary to induce significant cell death [21]–[25].

In this study, we examined the association of FAK overexpression with *FAK* copy number gain in OCCC patient samples and cell lines. We then tested the responses of ovarian cancer cell lines to FAK, PI3K/mTOR, and BCL-2/BCL-XL inhibitors either alone or in combination. We found that co-treatment with FAK and BCL-2/BCL-XL inhibitors had a synergistic effect on induction of apoptosis, and the down-regulation of MCL1 by FAK inhibition might contribute to this synergism.

## Materials and Methods

### Patient tissue samples and cell lines

A total of 67 formalin-fixed, paraffin-embedded (FFPE) samples from patients diagnosed with ovarian clear cell carcinoma were retrieved from the sample archives and were anonymized. This study was approved by the Institutional Review Board of the Samsung Medical Center by an informed consent waiver using the anonymized archival tissues with retrospective clinical data. Ovarian adenocarcinoma or clear cell adenocarcinoma cell lines were obtained from 3 different institutes: SNU-8 and SNU-119 from the Korean Cell Line Bank (KCLB) (Seoul, South Korea); SKOV3, A2780, ES2, RMGI, and TOV21G from the American Type Culture Collection (ATCC, Manassas, VA); and OVMANA, OIVSE, OVSAHO, and OVTOKO from the Health Science Research Resources Bank (HSRRB) (Tokyo, Japan). OCCC cell lines are OVMANA, RMGI, TOV21G, OVTOKO, ES2 and OVISE. TOV21G was cultured in a 1∶1 mixture of MCDB115 and R119 media supplemented with 10% serum and antibiotics (100 µg/mL penicillin and streptomycin). The other cell lines were cultured in RPMI-1640 medium supplemented with 10% fetal bovine serum and antibiotics.

### Immunohistochemistry of FAK expression

The slides were deparaffinized, followed by antigen retrieval. Endogenous peroxide and nonspecific proteins were blocked. The slides were then incubated with a FAK primary antibody (#05-537, Millipore, Billerica, MA) in a blocking solution overnight at 4°C. After washing, the horseradish peroxidase-conjugated secondary antibody was added. The slides were stained with DAB substrate and counterstained with hematoxylin. The expression of FAK was interpreted and graded. Scores of 0 or 1+ were considered negative and those of 2+ or 3+ were considered moderately or strongly positive for FAK overexpression.

### Estimation of FAK copy number with quantitative real-time polymerase chain reaction (PCR)

Genomic DNA of 67 formalin-fixed, paraffin-embedded (FFPE) samples was isolated using the QIAamp DNA FFPE Tissue Kit (Qiagene, Hilden, Germany) according to the manufacturer's protocol. Briefly, 10 ng of genomic DNA was amplified using the TaqMan® Universal Master Mix II and the ABI 7900 system (Applied Biosystems, Carlsbad, CA). The genomic DNA samples were denatured at 94°C for 10 min and then subjected to 40 cycles of denaturation at 94°C for 15 s and annealing and extension at 60°C for 1 min. We amplified 2 genomic regions of *FAK* and 3 silent genomic regions of *IL5*, *SLC41A2*, and *HOMEZ* as reference genes; these genes were identified through a previously conducted array-comparative genomic hybridization study. Primer sequences are listed in Table S1 in File S1. The relative FAK copy number log ratio was calculated by using the (-)ΔΔCT method. ΔCT_tumor_ and ΔCT_normal_ represents the difference between the average CTs of the FAK and the reference gene in the tumor and normal genomic DNA (4 normal blood samples). ΔΔCT was obtained by subtracting ΔCT_normal_ from ΔCT_tumor_. Samples with a copy number log ratio ((-)ΔΔCT) higher than 0.32 and 1.0 were considered copy number gain and positive for gene amplification, respectively.

### Mutation screening with Sanger sequencing

The same genomic DNA isolated from the 67 FFPE samples was used for mutation screening. Exons 9 and 20 of *PIK3CA* were PCR-amplified and were subjected to sequencing using the Sanger sequencing method. Primer sequences and PCR conditions will be provided upon request.

### Inhibition of FAK and PYK2 auto-phosphorylation of by FAK inhibitors

OVISE and SNU-8 cells were cultured until the cells reached 80% confluence in 100 mm dishes. The cells were then exposed to two FAK inhibitors (PF-562271 or PF-57322) at varying drug concentrations (0.01, 0.1, 1 or 10 µM) for 24 hr. The cells were lysed and subjected to Western blot analysis. The small-molecule compounds PF-562271 (PF271) and PF-573228 (PF228) were obtained from Selleck Chemicals (Houston, TX).

### Cell proliferation and cytotoxicity assay

Cells were seeded at a density of 1×10^4^ cells or 5×10^3^ cells (OVISE, ES2, SNU-119) in 96 well plates for the cell proliferation and viability tests. Drugs were added 1 day after the cells were seeded, and the cells were cultured for 3 days. To generate dose-response curves, the drugs (PF271, PF228, ABT-737, and sorafenib) were 2-fold serially diluted from 50 µM to 0.19 µM. ABT-737 or sorafenib was combined with 1 µM BEZ235 and/or 5 µM PF271 for the combination treatments. Cell proliferation and viability were measured using the WST-1 reagent according to the manufacturer's protocols (Roche, Indianapolis, IN). The optical density was measured at 450 nm and 600 nm 4 hr after adding the WST-1 reagent. The concentration required to reduce cell viability by 50% (EC_50_) was calculated using package drc (http://cran.r-project.org/web/packages/drc/) with the 3-parameter logistic function standard curve analysis for dose response. The cells were seeded at a density of 4×10^4^ cells in 96 well plates for the cytotoxicity test. Drugs, including ABT-737 (0.2 or 1 µM), BEZ235 (0.2 or 1 µM), and PF271 (1 or 5 µM)) were added individually or in combination on the next day and the cells were incubated for 24 hr. Cell cytotoxicity was measured using the Cytotoxicity Detection Kit (Roche) and was calculated with the following formula ([treatment − low control]/[high control − low control] ×100). The small-molecule compounds BEZ235, MK-2206, sorafenib, and ABT-737 were obtained from Selleck Chemicals (Houston, TX).

### Western blot analysis

OVMANA, RMGI, OVISE and TOV21G cells were treated individually or in combination with 1 µM ABT-737, 1 µM BEZ235 and/or 5 µM PF271 for 6 hr. For time course experiments, RMGI and OVISE cells were exposed to 5 µM or 10 µM PF271 or PF228 for 3, 6 and 24 hr. The drug-treated cells were lysed with M-PER buffer (Pierce Biotechnology, Rockford, IL) containing 1× protease and phosphatase inhibitor cocktail (Roche). Then, 40 µg of lysate was separated on 4–15% or 12% precast sodium dodecyl sulfate-polyacrylamide gels and transferred onto polyvinylidene fluoride membranes (Bio-Rad Laboratories, Hercules, CA). The membranes were blocked with 5% non-fat dry milk and incubated with appropriate primary and secondary antibodies. Signals were detected using the SuperSignal West Pico Chemiluminescent Substrate (Pierce Biotechnology). The following antibodies were used as follows: caspase-8 (9764), phospho-Akt (Ser473) (4060), Akt (pan) (4691), phospho-Pyk2 (Tyr402) (3291), Pyk2 (3480), PARP (9542), BCL-XL (2764), MCL1 (5453), caspase-3 (9668), Bcl-2 (4223), phospho-FAK (Cell Signaling Technologies, Beverly, MA); FAK (Millipore); GAPDH (sc-25778), and goat anti-rabbit IgG (sc-3837) (Santa Cruz Biotechnology, Santa Cruz, CA).

### Fluorescence-activated cell sorting (FACS) analysis

RMGI, OVISE and TOV21G cells were treated individually or in combination with 1 µM ABT-737, 1 µM BEZ235 and/or 5 µM PF271 for 6 hr. Drug-treated cells were trypsinized and counted. Then, 1.5×10^6^ cells were washed with cold 1×PBS, fixed with 70% cold ethanol for propidium iodide (PI) staining. The cells were washed with 1×PBS, incubated with staining solution including 50 µg/ml PI and 200 µg/ml RNase A for 15 min at 37°C and analyzed using a Becton-Dickinson FACS Calibur flow cytometer.

### Statistical analysis

The significance of the correlation between FAK expression and FAK copy number was determined with Fisher's exact test. P values less than 0.05 were considered to indicate significance. The EC_50_ values were calculated using the drc package (http://cran.r-project.org/web/packages/drc/) with the 3-parameter logistic function standard curve analysis for dose response. Synergism was determined with a two-way analysis of variance [26]

## Results

### *FAK* copy number gains underlie FAK overexpession in OCCCs, but do not correlate with *PIK3CA* mutations

**Figure 1:**
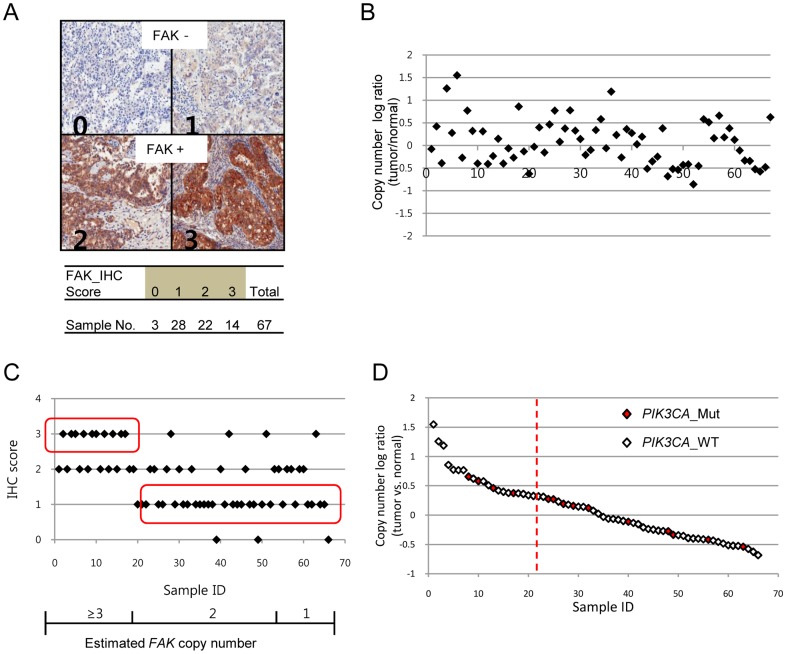
FAK overexpression was associated with an increased copy number in OCCCs. A, Paraffin-embedded tumor sections were immunohistochemically stained using an anti-FAK antibody. The FAK staining results were scored into four categories based on the signal intensity: 0, no detection; 1, weak; 2, moderate; 3, strong. B, Variation in the FAK copy number was assessed with quantitative PCR. Copy number log ratios higher than 0.32 and 1.00 were considered evidence of copy number gain and gene amplification, respectively. The X-axis represents the samples. C, FAK overexpression (IHC score 3) correlated with the gains in copy number (copy number log ratio >0.32). Weak expressions of FAK (IHC score 1) resulted in normal or reduced FAK copy numbers (copy number log ratio <0.32). D, Samples with a mutation in *PIK3CA* (*PIK3CA*_Mut) are marked as red diamonds on the plot. The vertical dotted line indicates a copy number log ratio of 0.32.

We first examined FAK protein expression levels in OCCC samples. More than 50% of the OCCC samples were positive for FAK expression, and about 21% of them showed strong FAK expression (Fig. 1A). We then assessed whether FAK overexpression was due to *FAK* copy number gain in OCCCs. *FAK* copy number gains and losses were estimated to have frequencies of 31% and 28% in the OCCC samples, respectively (Fig. 1B, Table S2 in File S1). In particular, 3 cases (4%) showed high level copy number gain, which were considered gene amplifications. The majority of the altered samples showed one copy gain or loss. *FAK* copy number gains significantly correlated with the strong protein expression (Fig. 1C, p-value = 0.0001). This suggests that *FAK* could be a selective target in a subset of OCCCs during tumorigenesis or for tumor progression. The fact that mutations in *PIK3CA* are common in OCCCs [2], [3] and that activated FAK sends signals via the PI3K/AKT signaling pathway suggests that *PIK3CA* mutations and FAK copy number gains might be mutually exclusive. Therefore, we screened the samples for *PIK3CA* mutations, which were found in 20% of the samples tested (Fig. 1D). *PIK3CA* mutations were found in some samples with a *FAK* copy number gain, suggesting that *FAK* copy number gains were not strictly mutually exclusive to *PIK3CA* mutations. Nonetheless, about 40% of the samples showed *FAK* copy number gain or *PIK3CA* mutation. This suggests that the FAK/PI3K/AKT axis might play an important role in OCCCs.

### Ovarian cancer cell lines are more sensitive to dual FAK and PYK2 inhibitors

**Figure 2:**
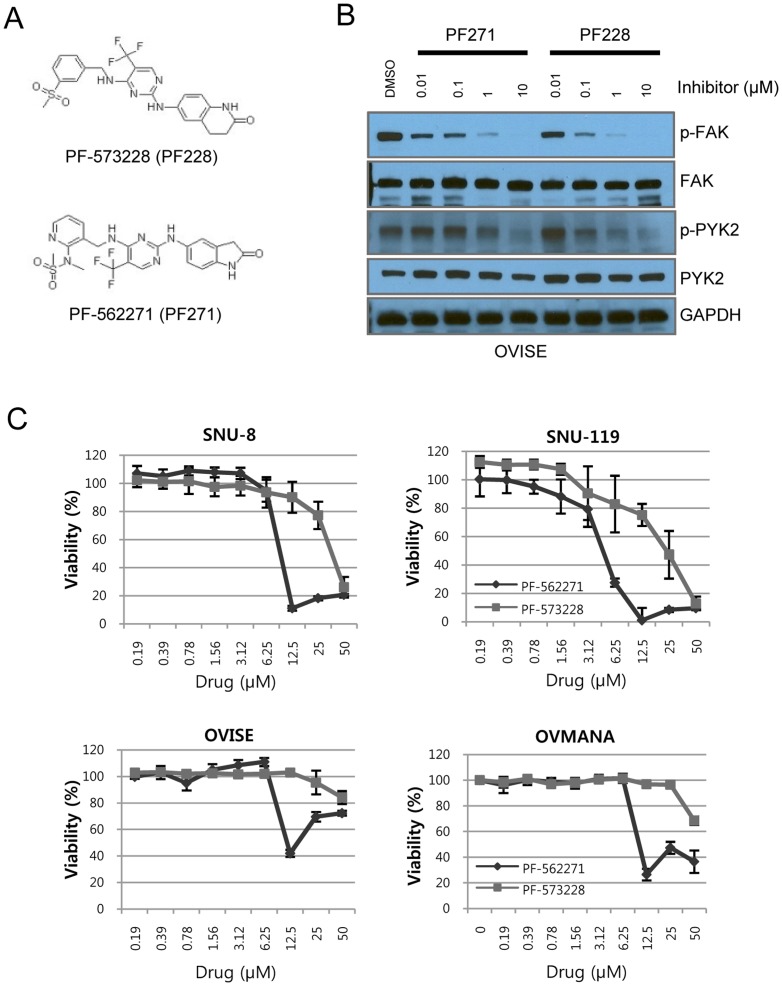
Drug responses of ovarian cancer cell lines to the FAK inhibitors. A, The chemical structure of the FAK inhibitors PF-573,228 (PF228) and PF-562,271 (PF271). B. OVISE cells were incubated for 24 hr at the indicated concentrations of the FAK inhibitors. Immunoblots were performed to assess inhibition of auto-phosphorylation by the FAK inhibitors. A vehicle control, containing dimethyl sulfoxide (DMSO), was performed. C, The viability of the ovarian cancer cells was determined after exposure to PF228 and PF271 for 72 hr. The results from only one experiment are shown; two additional studies also exhibited equivalent results.

Once we characterized ovarian cancer cell lines (Fig. S1, Tables S3 and S4 in File S1), we treated the cancer cell lines with the FAK inhibitors, PF-573,228 (PF228) and PF-562,271 (PF271) (Fig. 2A). PF228 is a selective inhibitor of FAK [27], whereas PF271 is a dual inhibitor of FAK and PYK2 [28]. However, both drugs were able to inhibit auto-phosphorylation at Y397 of FAK and Y402 of PYK2 dose-dependently in OVISE cells (Fig. 2B). The auto-phosphorylation of FAK was inhibited at concentrations lower than 0.1 µM in SNU-8 cells (Fig. S2). Most of the ovarian cancer cell lines were more sensitive to PF271 than to PF228 (Fig. 2C). The SNU-119 and SNU-8 cell lines were 4-fold more sensitive to PF271 than to PF228 (Fig. 2C). For both drugs, the lowest EC_50_ (PF228: 19.1 µM; PF271: 4.41 µM) was observed in SNU-119 cells, which showed a high level of copy number gains for FAK (Fig. S1 and Table S3 in File S1). However, the EC_50_ of SNU-8 cells (PF228: 27.3 µM; PF271: 6.79 µM), which showed a 1 copy number loss, was as low as that of SNU-119 cells. Although the *FAK* copy number and protein levels of OVMANA and OVISE cells were higher than those of SNU-8 cells (Fig. S1 and Table S3 in File S1), the OVMANA and OVISE cell lines seemed to be more resistant to PF271 and PF228 than did SNU-8 cells (Fig. 2C). As a result, cancer cells are much more sensitive to PF271 than PF228. However, sensitivity to PF271 or PF228 was not significantly associated with FAK copy number, suggesting that other factors might be involved.

### The EC_50_ of ABT-737 is lower when combined with PF271

**Figure 3:**
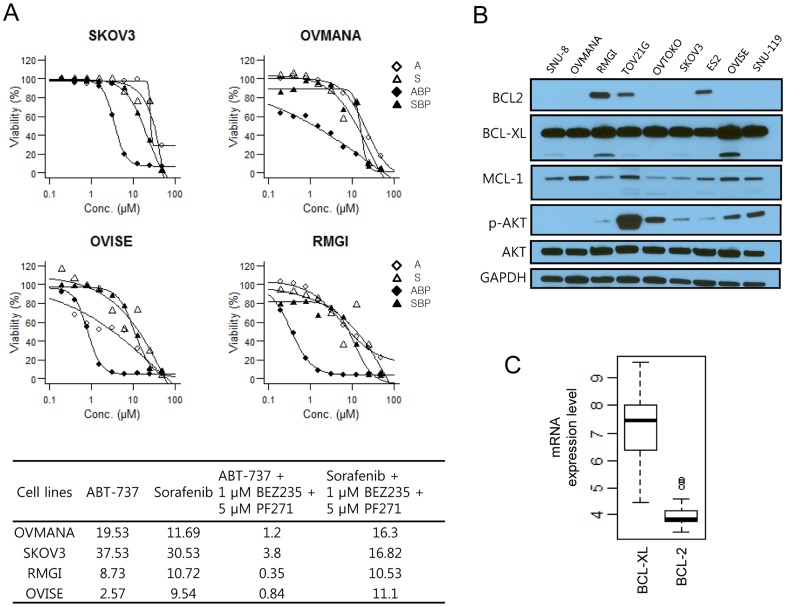
PF271 enhanced the lethality of ABT-737. A-upper panels, Ovarian cancer cell lines were exposed to various concentrations of ABT-737 (⋄: A), sorafenib (△: S), ABT-737 in combination with 1 µM BEZ235 and 5 µM PF271 (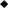: ABP), or sorafenib in combination with 1 µM BEZ235 and 5 µM PF271 (▴: SBP) for 72 hr. Cell viability (%) was calculated and dose response curves were predicted with a three-parameter log-logistic function. A-lower panel, The EC_50_ of the anti-apoptotic inhibitors alone or in combination. This represents the result of one experiment; two additional studies also exhibited equivalent results. B, The protein expression patterns of the anti-apoptotic proteins and the phosphorylation status of AKT in nine ovarian cancer cell lines. Immunoblots were done to assess the basal expression levels of BCL-2, BCL-XL, MCL1, and phosphorylated AKT. C, The standard mRNA expression levels of BCL-XL and BCL-2 in forty ovarian cancer cell lines (CCLE database).

To identify molecular targeted agents that exhibit a synergistic effect when co-administered with PF271, we treated cells with PF271 in combination with other inhibitors that target genes, particularly involved in the cell survival and apoptotic pathways such as the allosteric AKT inhibitor MK-2206, the PI3K/mTOR inhibitor BEZ235, the multiple kinase inhibitor sorafenib, and the BCL-2/BCL-XL inhibitor ABT-737. Drug combinations of PF271/MK-2206 or PF271/BEZ235 exhibited minor effects (data not shown). However, the combination of PF271/BEZ235/ABT-737 profoundly reduced cell growth and cell viability compared to the ABT-737 alone treatment (Fig. 3A). Sorafenib is known to down-regulate MCL1 [29]. Co-treatment of PF271/BEZ235 with sorafenib exhibited no significant effect compared to sorafenib alone (Fig. 3A). Notably, the RMGI and OVISE cell lines were at least 2-fold more sensitive to ABT-737 than the OVMANA and SKOV3 cell lines (Fig. 3A).

To gain insight into the mechanism underlying the distinct sensitivities to the drug combinations described above, we performed Western blot analysis for anti-apoptotic proteins, and phospho-AKT. The BCL-2 protein was expressed in RMGI, TOV21G, and ES2 cells, but not in other cell lines (Fig. 3B). The BCL-XL protein was highly expressed in most cells and its expression was slightly increased in RMGI and OVISE cells compared with other cells (Fig. 3B). We analyzed the mRNA levels of BCL-2 and BCL-XL in all ovarian cancer cell lines obtained from cancer cell line encyclopedia (CCLE) (Fig. 3C). In agreement with our results, the BCL-XL expression level was much higher than that of BCL-2. In most of the cells, BCL-2 did not appear to be expressed (Fig. 3C). This finding indicates that BCL-XL is the major target of ABT-737, at least in ovarian cancer cells.

This result suggests that inhibition of FAK required simultaneous inhibition of the anti-apoptotic proteins BCL-2/BCL-XL for reducing cell viability. BCL-XL, rather than BCL-2, might be responsible for the enhanced sensitivity of ovarian cancer cell lines to ABT-737.

### Synergistic effect of PF271 and ABT-737 in triggering cell death

**Figure 4:**
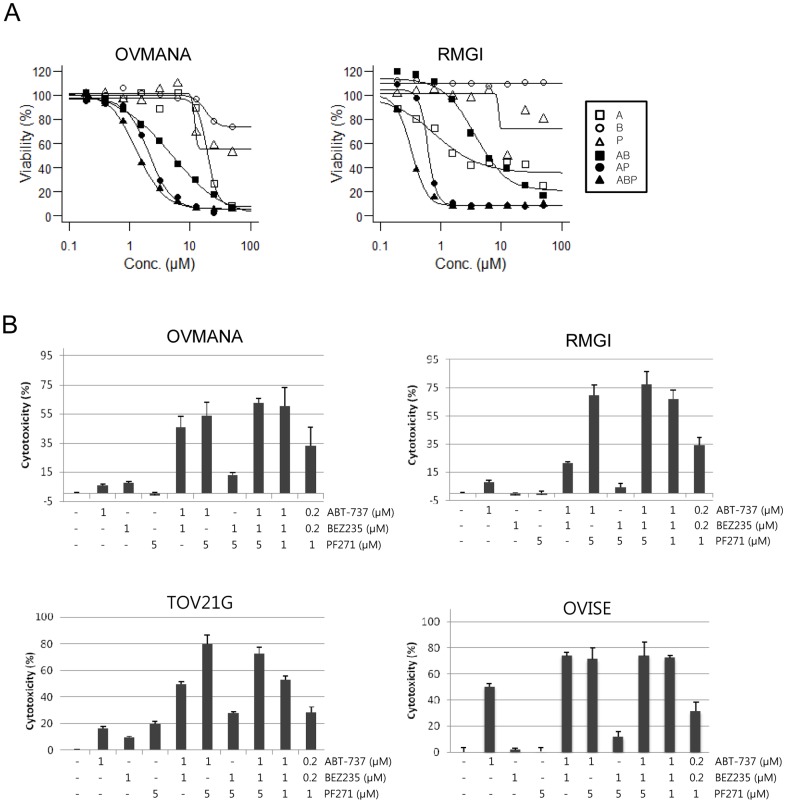
Synergistic effect of the FAK and BCL2/BCL-XL inhibitors in triggering cell death. A, Cell proliferation assays were performed 72-737 (□: A), BEZ235 (○: B), PF271 (△: P), ABT-737 combined with 1 µM BEZ235 (▪: AB), ABT-737 combined with 5 µM PF271 (•: AP), and ABT-737 combined with 1 µM BEZ235 and 5 µM PF271 (▴: ABP) for 72 hr. Cell viability (%) was calculated and dose response curves were predicted with a four-parameter log-logistic function. B, OVMANA, RMGI, TOV21G, and OVISE cells were exposed to ABT-737 (0.2 or 1 µM), BEZ235 (0.2 or 1 µM), or PF271 (1, or 5 µM) individually or in combinations for 24 hr. The values of cytotoxicity (%) indicate the percentage of dead cells for each treatment. The cytotoxicity data represented in B are the means ± SD from a single representative of three experiments. A synergistic effect was observed when the cells were treated with both ABT-737 and PF271.

To further evaluate the effects of combining PF271 and ABT-737, ovarian cancer cell lines were treated with PF271, ABT-737, and BEZ235 alone or in combination of either PF271 (5 µM), BEZ235 (1 µM), or PF271 (5 µM)/BEZ235 (1 µM) and increasing doses of ABT-737, and dose-response curves were determined. In particular, RMGI cells were relatively sensitive to ABT-737 alone, but were resistant to BEZ235 as compared to OVMANA cells (Fig. 4A). As shown in Fig. 3, treatment with PF271 and ABT-737 lowered the EC_50_ by more than 6-10-fold in RMGI and OVMANA cells (Fig. 4A). In the RMGI cell line, the combination of PF271 and ABT-737 further lowered the EC_50_ as compared with the combination of BEZ235 and ABT-737 (Fig. 4A).

We then tested the cytotoxic effect of 24 hr exposure to a fixed dose alone, or in double or in triple combinations. In RMGI and OVMANA cells, ABT-737 (1 µM), PF271 (5 µM), or BEZ235 (1 µM) alone had little effects on cytotoxicity. However, co-treatment with ABT-737/PF271, ABT-737/BEZ235, or ABT-737/BEZ235/PF271 markedly increased cytotoxicity, indicating a synergistic drug effect (Fig 4B and Fig. S3). A 5-fold lower drug concentration of ABT-737/BEZ235/PF271 could still effectively to induce a synergistic effect in OVMANA and RMGI cells (Fig. 4B). PF271/BEZ235 co-treatment had little effect on cytotoxicity. Of note, co-treatment with ABT-737/PF271 induced a greater synergistic effect on cytotoxicity compared with the ABT-737/BEZ235 combination, particularly in the RMGI and TOV21G cell lines (Fig. 4). Therefore, we confirmed that the ABT-737/PF271 combination synergistically induce cell death.

### Enhancing apoptosis and the degradation of BCL-XL and MCL1

**Figure 5:**
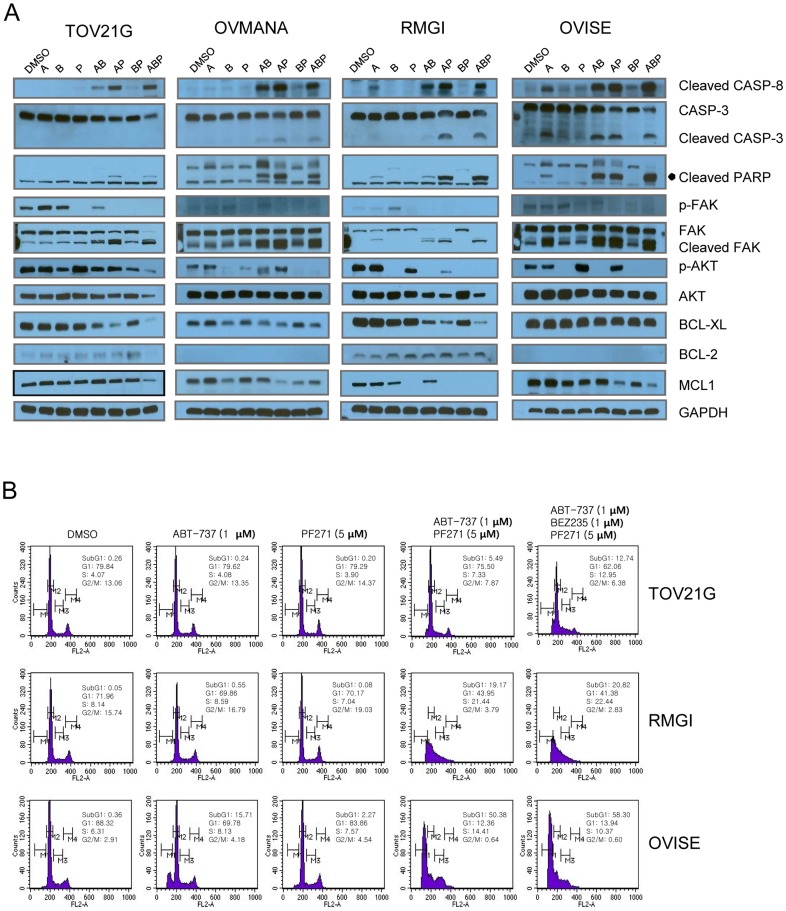
FAK and BCL2/BCL-XL inhibitors induced apoptosis. A, OCCC cell lines were treated with 1 µM ABT-737 (A), 1 µM BEZ235 (B), or 5 µM PF271 (P), individually or in combinations (AB indicates ABT-737/BEZ235; AP, ABT-737/PF271; BP, BEZ235/PF271; ABP, ABT-737/BEZ235/PF271) at the same drug concentrations for 6 hr. Cell lysates were prepared and subjected to Western blot analysis using the indicated antibodies. Cleavage of caspase-3 (CASP3), caspase-8 (CASP8), PARP, and FAK indicated that apoptosis had occurred. BCL-2 was not expressed in OVMANA and OVISE. B, TOV21G, RMGI and OVISE cells were treated individually or in combination with 1 µM ABT-737, 1 µM BEZ235 and/or 5 µM PF271 for 6 hr. The cells were fixed with 70% ethanol, were stained with propidium iodide (PI) and analyzed using a FACS Calibur flow cytometer.

To investigate the synergistic mechanism by which ABT-737 and PF271 induce apoptosis, we treated cells with ABT-737, PF271, and BEZ235 alone or in combinations for 6 hr (Fig. 5) and analyzed the cell lysates and cell cycle. Dramatic increases in the levels of cleaved caspase-8, caspase-3, PARP, and FAK, which are markers of the activation of apoptosis, were observed in the cells co-treated with ABT-737 and either PF271 or BEZ235, but not with PF271 and BEZ235 alone (Fig. 5A), which is in agreement with the result of the cytotoxicity test (Fig. 4B). ABT-737 alone induced to a minor extent cleavage of caspase-3 and caspase-8, PARP, and FAK in RMGI and OVISE cells, which seemed to be sensitive to ABT-737, but not in TOV21G and OVMANA cells (Fig. 5A). Interestingly, the levels of cleaved caspase-8 and PARP were correlated with the differential cytotoxicity (Figs. 4 and 5A), but the levels of p-AKT were not.

The synergistic induction of apoptosis by co-treatment with ABT-737 and PF271 was also assessed with fluorescence-activated cell sorting (FACS) analysis (Fig. 5B). Consistent with the Western blot result (Fig. 5A), The number of apoptotic cells (subG1) greatly increased when the cells were treated in combination. The number of subG1 cells were much lower in TOV21G cells than in RMGI and OVISE cells, which indicated that TOV21G cells were relatively resistant to the induction of apoptosis by ABT-737 and PF271 as shown in Fig. 5A.

BCL-XL was markedly degraded in the co-treated TOV21G and RMGI cells, whereas BCL-2 was not much changed (Fig. 5A). In agreement with reports involving other cell types [30], BEZ235 treatment, which interrupts the PI3K/AKT pathway, resulted in decreased AKT phosphorylation and MCL1 protein levels in OVMANA, RMGI and OVISE cells. MCL1 was not clearly down-regulated in TOV21G cells where AKT phosphorylation was moderately down-regulated by BEZ235. Most likely, mutations in *PIK3CA* and *KRAS* maintained sustained AKT phosphorylation in TOV21G cells (Table S4 in File S1). These results suggested that the PI3K/AKT pathway might regulate the MCL1 levels in OCCC cell lines. MCL1 was apparently down-regulated in RMGI and OVISE cells by treatment with PF271 alone. In the OVMANA cells, the MCL1 level was decreased by co-treatment with PF271 and ABT-737, but not by either PF271 or ABT-737 alone. Unlike BEZ235, p-AKT levels were not concordant with MCL down-regulation. This might result from the adaptive activation of the FAK downstream effector genes. Although whether PF271 down-regulates MCL1 via the PI3K/AKT pathway is not clear, this finding suggested the potential that PF271 might down-regulate MCL1.

Collectively, co-treatment with PF271 and ABT-737 strikingly induced apoptosis in OCCC cells, which was associated with the down-regulation of either BCL-XL or MCL1 or both.

### Down-regulation of MCL1 by FAK inhibition

**Figure 6:**
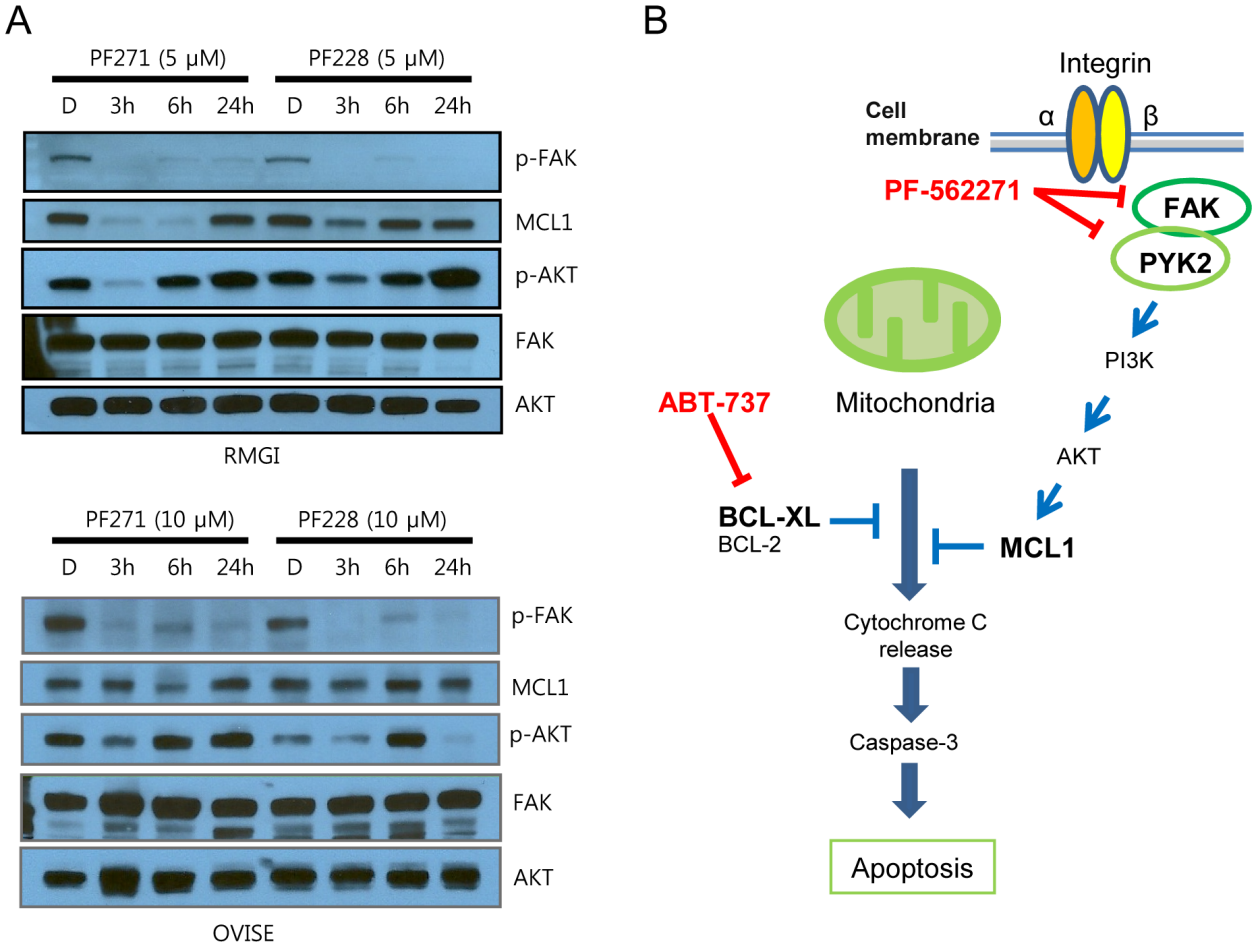
FAK inhibitors down-regulate MCL1. A, MCL1 was transiently down-regulated by treatment with PF271 or PF228. RMGI and OVISE cells were incubated in the presence of PF271 or PF228 at 5 µM for RMGI and 10 µM for OVISE. The cells were harvested at the indicated time points and lysed. D indicates the dimethyl sulfoxide (DMSO) vehicle control. Immunoblots were performed for p-FAK, FAK, MCL1, AKT, and p-AKT. B, A proposed schematic model by how the combined pharmacological inhibition of FAK/PYK2 and BCL-XL/BCL-2 induces apoptosis.

We assumed that PF271 may transiently down-regulate MCL1 through the PI3K/AKT pathway. To test this hypothesis, we performed a time-course experiment. We treated RMGI and OVISE cells with PF271 or PF228 and harvested the cells at 3, 6, and 24 hr after drug treatment. MCL1 was markedly reduced at 3 and 6 hr after PF271 treatment (Fig. 6A). Likewise, the MCL1 level was slightly decreased at 3 hr and recovered at 6 hr after PF228 treatment (Fig. 6A). FAK inhibitors also suppressed AKT phosphorylation, which correlated with MCL1 down-regulation. Therefore, these data suggested that both FAK inhibitors can down-regulate MCL1 transiently through the PI3K/AKT pathway, and PF271 differs from PF228 with respect to the degree and duration of MCL1 reduction.

## Discussion

In this study, we found that co-treatment with PF271 and ABT-737 effectively induced apoptosis, and FAK inhibition resulted in the transient down-regulation of MCL1. Therefore, co-treatment with PF271 and ABT-737 led to the inhibition of the anti-apoptotic proteins MCL1 and BCL-XL/BCL-2 simultaneously (Fig. 6B). PF271 alone or in combination with ABT-737 down-regulated MCL1 in OCCC cell lines, most strikingly in RMGI cells (Figs. 5 and 6). Another FAK inhibitor, PF228, was also able to down-regulate MCL1. Therefore, these results argue that FAK inhibition leads to MCL1 down-regulation. The differences in the rate and duration of the MCL1 reduction between PF271 and PF228 (Fig. 6A) might underlie the differences in the sensitivity of ovarian cancer cells to both drugs, as shown in Fig. 2. It has been reported that down-regulation of MCL1 renders cancer cells susceptible to anoikis [31], [32]. Woods et al. observed that following detachment of the cells from the culture plate, MCL1 was rapidly degraded through a glycogen synthase kinase 3β (GSK-3B)–mediated proteasomal pathway [32]. When the cells are detached, FAK is dephosphorylated [5], which causes cells to undergo anoikis eventually. PF271 is an ATP competitor that inhibits the auto-phosphorylation of FAK ([28] and Fig. 2). Therefore, PF271 treatment might imitate detachment in cells.

The half-life of MCL1 is short [33]. FAK downstream pathways such as the MEK/ERK1/2 or PI3K/AKT pathways can regulate the half-life of MCL1 at the transcriptional or translational levels. In particular, activation of ERK1/2 promotes the transactivation of MCL1 mRNA or inhibits the turnover of MCL1 protein by phosphorylation at T163 [34]. The PI3K/AKT pathway inhibits MCL1 proteasomal degradation indirectly, which is promoted by activated GSK3 [35]. In RMGI cells, p-AKT and MCL1 levels were commonly reduced following PF271 treatment and then recovered (Fig. 6A), suggesting that MCL1 is controlled by the FAK/PI3K/AKT pathway. The rate of MCL1reduction varied among the cell lines. TOV21G and OVMANA cells were more likely to be resistant to the reduction, whereas RMGI and OVISE cells were relatively sensitive. Oncogenic mutations in effector genes downstream of FAK might contribute to the resistance. TOV21G cells harbor oncogenic mutations in *KRAS* and *PIK3CA*, which are involved in both the PI3K/AKT and RAS/RAF pathways. OVMANA cells have an oncogenic *PIK3CA* mutation, whereas RMGI and OVISE cells do not have mutations in *KRAS* and *PIK3CA*. Oncogenic mutations in both signaling pathways might attenuate upstream death signals such as anoikis in OCCC cell lines.

We observed greatly accelerated apoptosis when PF271 was combined with ABT-737, but not when it was combined with sorafenib, the AKT inhibitor MK-2206, or the PI3K/mTOR inhibitor BEZ235. Given that these targeted agents inhibit multiple kinases, including B-RAF, PI3K, and AKT, they might mainly contribute to MCL1 repression. These effects overlap with the inhibition of FAK by PF271. None of these agents may significantly affect the anti-apoptotic function of BCL-2/BCL-XL, even when in combination treatment. However, PF271 in combination with the BCL-2/BCL-XL inhibitor ABT-737 contributed to the simultaneous inhibition of both anti-apoptotic proteins MCL1 and BCL-2/BCL-XL. It has been reported that that the overexpression of MCL1 confers resistance to BCL-2/BCL-XL repression and vice versa [21]–[25]. Therefore, the simultaneous targeting of MCL1 and BCL-2/BCL-XL is required to increase the apoptotic cell death of tumor cells. Multiple mechanisms to either directly or indirectly decrease the MCL1 level have been reported. Our dual inhibition of FAK and PYK2 is an addition to the list of approaches for targeting ovarian cancer cells as well as other types of cancers in which FAK or PYK2 are overexpressed.

In addition to MCL1, other factors might play roles in enhancing the apoptosis induced by ABT/PF271. The cooperative activation of caspase-8, which is involved in the extrinsic apoptotic signaling pathway, could be one of the enhancing factors that contribute to the effect of ABT/PF271. Other groups and ours observed that ABT-737 alone can activate caspase-9 and caspase-3, as well as caspase-8 [25], [36], [37], with limited efficacy. Overexpressed FAK down-regulated procaspase-8 expression, which subsequently inhibited the downstream apoptosis pathway in HL-60/FAK cells [38]. Apoptosis caused by FAK inhibition required caspase-8 [39], Fas-associated death domain protein (FADD) [39], and death domain kinase receptor-interacting protein (RIP) [40], a major component of the death receptor complex, suggesting its role in the extrinsic pathway of apoptosis. Therefore, the enhanced sensitivity to ABT-737/PF271 might be mediated by cooperative caspase-8 activation, as a result of cross-talk between the extrinsic and intrinsic apoptotic pathways.

In conclusion, FAK copy number gains and *PIK3CA* mutations account for about 40% of OCCCs. Pharmacological inhibition of FAK and PYK2, particularly by PF271, led to the transient down-regulation of MCL1. Oncogenic mutations in downstream genes of FAK might attenuate the rate and duration of MCL1 repression, causing drug resistance. Co-treatment with PF271 and the BCL-2/BCL-XL antagonist ABT-737 was profoundly effective in inducing apoptosis. In addition to the inhibition of MCL1 and BCL-2/BCL-XL, both drugs might cooperate to activate the extrinsic and intrinsic apoptotic pathways, resulting in a further reduction of the apoptotic threshold. Our results can be applied to many other types of cancers such as lymphoma, breast, colon, and gastric cancers, where FAK and/or BCL-XL/BCL-2 are overexpressed.

## Supporting Information

Figure S1 **FAK copy number and protein levels in ovarian cancer cell lines.** A, FAK copy number log ratio (tumor vs. normal) were determined with quantitative real-time PCR. High level copy number gain was seen in the SNU-119 ovarian adenocarcinoma cell line and low level copy number gains in the SKOV3, OVSAHO, ES2, and OVISE cell lines. Copy number loss, probably 1 copy, was seen in the cell lines SNU-8 and OVMANA. Our copy number estimates were highly correlated with those downloaded from Cancer Cell Line Encyclopedia (CCLE) database (>95%, Supplemental Table 3). B, Western blottings were done to examine basal expression levels of FAK and PYK2 in 9 ovarian cancer cell lines. PYK2 was highly expressed in the OVTOKO, OVMANA, and OVISE cell lines. C, Each band intensity of FAK immunoblot result (B) was quantified and normalized with GAPDH. Normalized FAK levels (x-axis) were correlated with FAK copy number log ratios (Pearson correlation coefficient r = 0.737).
(TIF)

Figure S2 **Inhibition of FAK phosphorylation by FAK inhibitors in ovarian cancer cell lines.** SNU-8 (A) and TOV21G (B) cells were incubated for 24 hr in the presence of FAK inhibitors (PF271 or PF228) at the indicated concentrations (0.01–10 µM). FAK, PYK2, phosphorylated FAK (p-FAK, Y397) and PYK2 (p-PYK2, Y402) protein levels were determined by Western blot. GAPDH was served as a loading control. A vehicle control was performed, containing just dimethyl sulfoxide (DMSO).
(TIF)

Figure S3 **Synergistic effect of ABT-737 and PF271 on inducing cell death.** RMGI (A) and TOV21G (B) cells were exposed to decreasing doses of ABT-737 (A: 1, 0.5, 0.25, and 0.13 µM), PF271(P: 5, 2.5, 1.25, and 0.63 µM), or combinations (A+P) of the two agents at a fixed (1∶5) ratio. After a 24-hr exposure, cytotoxicity (%) was determined by measuring the activity of released lactate dehydrogenase (LDH) in culture media using Cytotoxicity Detection Kit. Data represent mean with ***standard deviation*** (n = 3).
(TIF)

File S1 **Supporting information.**
(DOC)
