# Supplementary figures and images for: Targeted Inhibition of FAK, PYK2 and BCL-XL Synergistically Enhances Apoptosis in Ovarian Clear Cell Carcinoma Cell Lines
Source: PLoS One. 2014 Feb 11;9(2):e88587. doi: 10.1371/journal.pone.0088587 (PMC3921183; doi:10.1371/journal.pone.0088587)

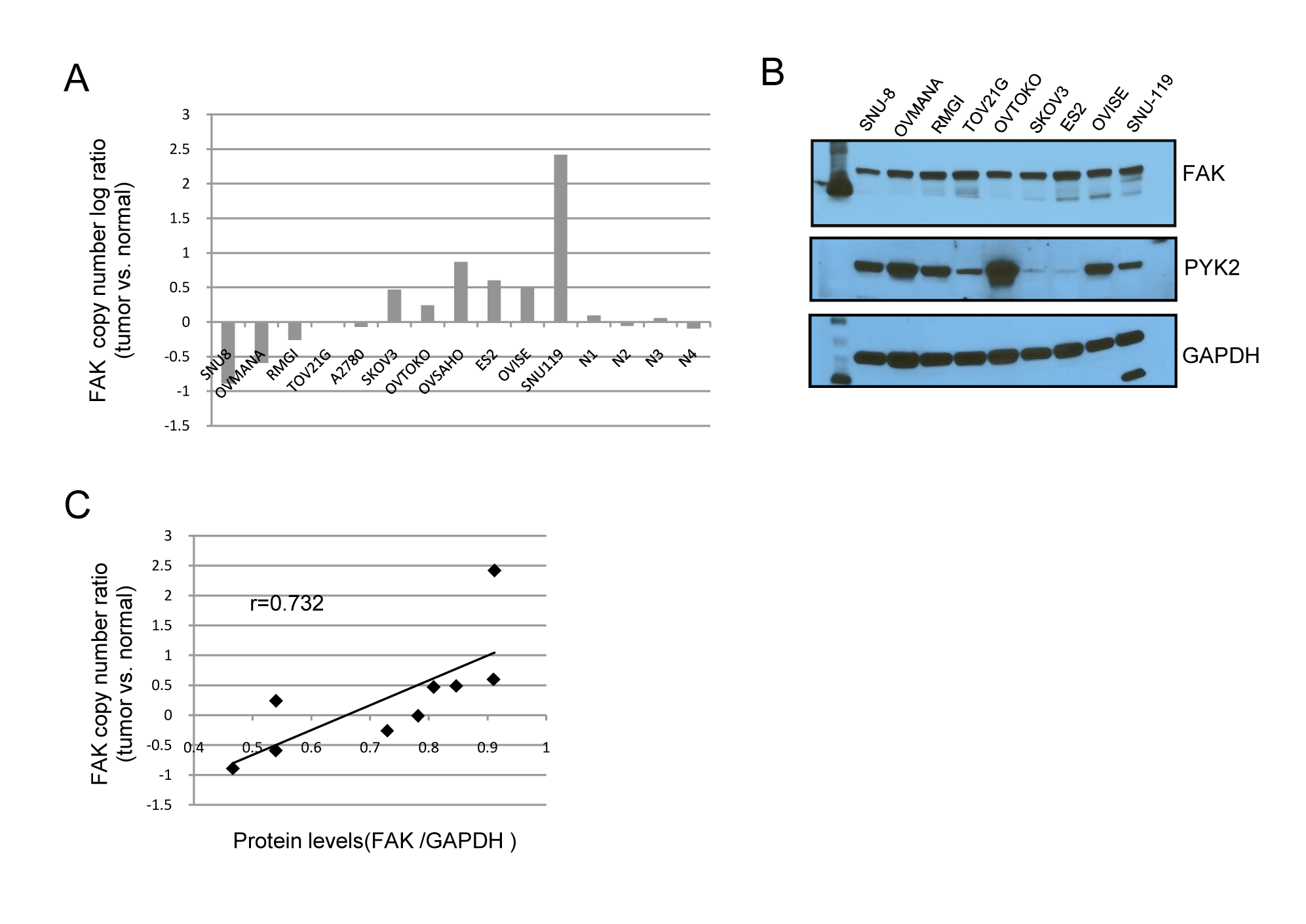

Supplement: Figure S1 — FAK copy number and protein levels in ovarian cancer cell lines. A, FAK copy number log ratio (tumor vs. normal) were determined with quantitative real-time PCR. High level copy number gain was seen in the SNU-119 ovarian adenocarcinoma cell line and low level copy number gains in the SKOV3, OVSAHO, ES2, and OVISE cell lines. Copy number loss, probably 1 copy, was seen in the cell lines SNU-8 and OVMANA. Our copy number estimates were highly correlated with those downloaded from Cancer Cell Line Encyclopedia (CCLE) database (>95%, Supplemental Table 3). B, Western blottings were done to examine basal expression levels of FAK and PYK2 in 9 ovarian cancer cell lines. PYK2 was highly expressed in the OVTOKO, OVMANA, and OVISE cell lines. C, Each band intensity of FAK immunoblot result (B) was quantified and normalized with GAPDH. Normalized FAK levels (x-axis) were correlated with FAK copy number log ratios (Pearson correlation coefficient r = 0.737). (TIF) [file pone.0088587.s001.tif]

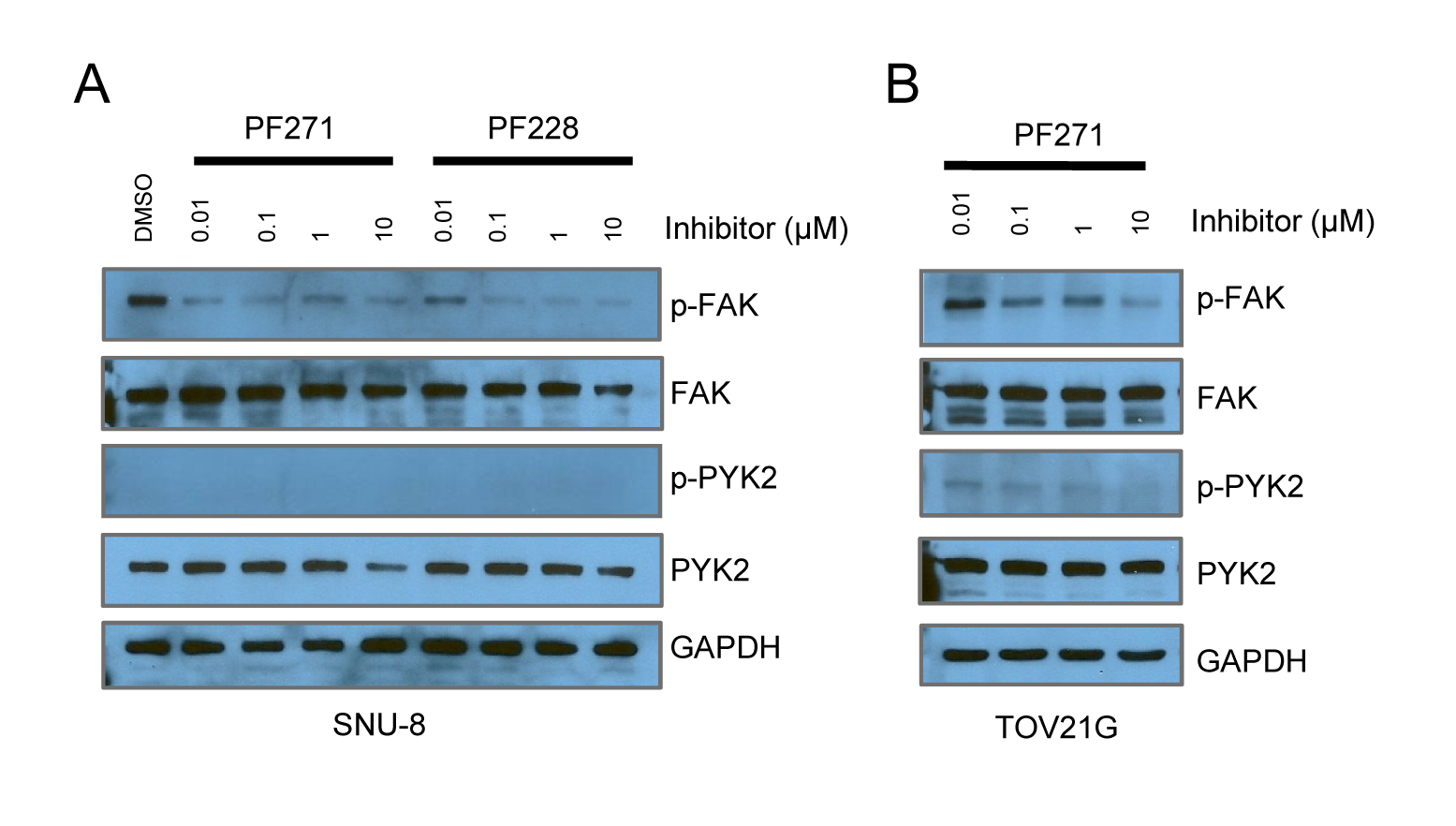

Supplement: Figure S2 — Inhibition of FAK phosphorylation by FAK inhibitors in ovarian cancer cell lines. SNU-8 (A) and TOV21G (B) cells were incubated for 24 hr in the presence of FAK inhibitors (PF271 or PF228) at the indicated concentrations (0.01–10 µM). FAK, PYK2, phosphorylated FAK (p-FAK, Y397) and PYK2 (p-PYK2, Y402) protein levels were determined by Western blot. GAPDH was served as a loading control. A vehicle control was performed, containing just dimethyl sulfoxide (DMSO). (TIF) [file pone.0088587.s002.tif]

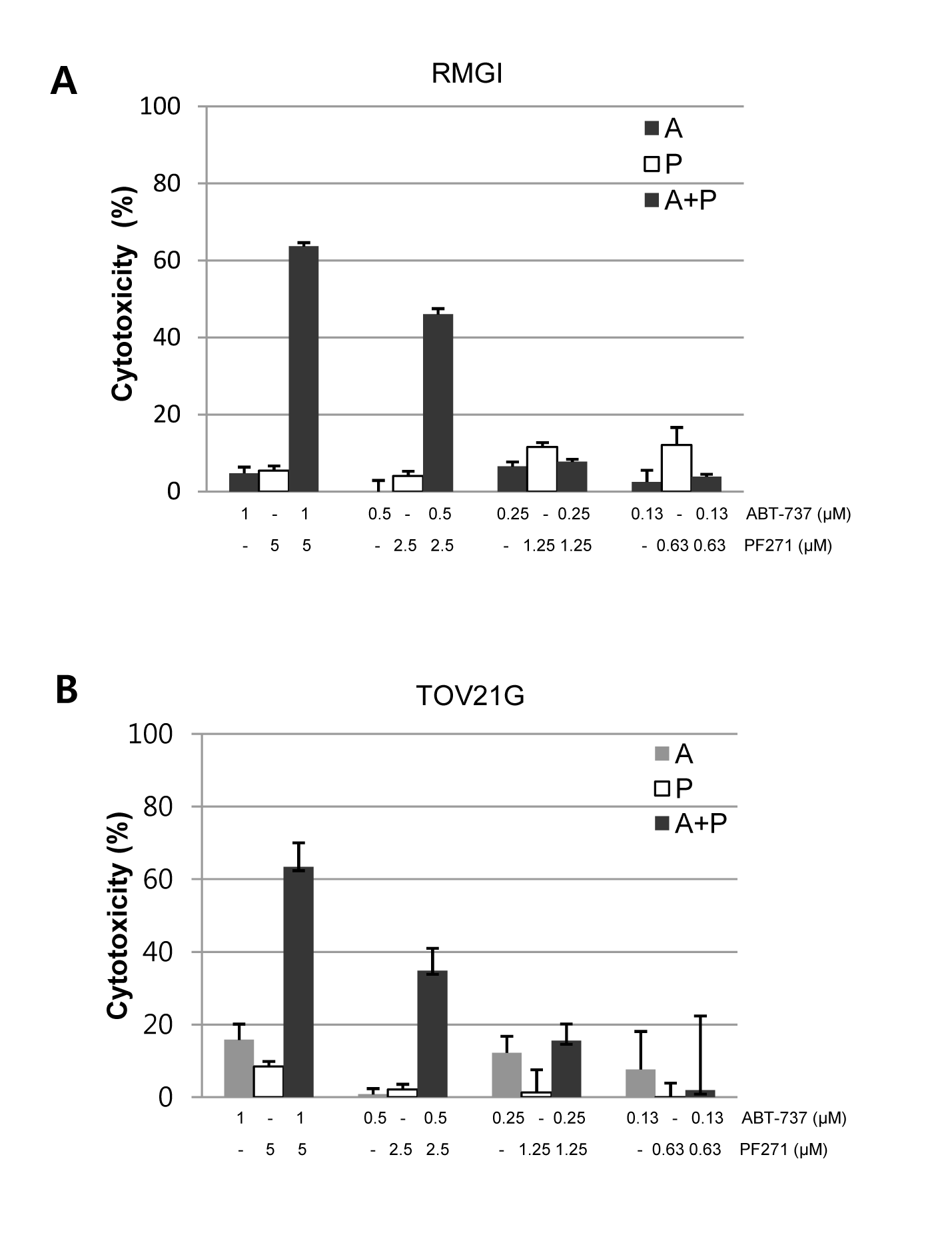

Supplement: Figure S3 — Synergistic effect of ABT-737 and PF271 on inducing cell death. RMGI (A) and TOV21G (B) cells were exposed to decreasing doses of ABT-737 (A: 1, 0.5, 0.25, and 0.13 µM), PF271(P: 5, 2.5, 1.25, and 0.63 µM), or combinations (A+P) of the two agents at a fixed (1∶5) ratio. After a 24-hr exposure, cytotoxicity (%) was determined by measuring the activity of released lactate dehydrogenase (LDH) in culture media using Cytotoxicity Detection Kit. Data represent mean with standard deviation (n = 3). (TIF) [file pone.0088587.s003.tif]
