# Supplementary material for: Targeted Inhibition of FAK, PYK2 and BCL-XL Synergistically Enhances Apoptosis in Ovarian Clear Cell Carcinoma Cell Lines
Source: PLoS One. 2014 Feb 11;9(2):e88587. doi: 10.1371/journal.pone.0088587 (PMC3921183; doi:10.1371/journal.pone.0088587)
Supplement: File S1 — Supporting information. (DOC) [file pone.0088587.s004.doc]

Table S1. Primer and TaqMan probe sequences used to assess FAK copy number alterations with quantitative real-time PCR.

| IL5-fw | GCAGTGCCAAGGTCTCTTTC |
| --- | --- |
| IL5-rev | TTGGAGCTGCCTACGTGTATG |
| IL5-probe | CAATGCACTTGTGGGAATTTCTGTGGG |
|  |  |
| HOMEZ -fw | GAATAGGACGCAACACTAGGAAC |
| HOMEZ -rev | CCGTACCCAGCCATACTAAGAG |
| HOMEZ -probe | ACTCAGGTTCTCAGTTTCCTGGTTGGT |
|  |  |
| SLC41A2-fw | GCCTCACCTCACAGGAATCAAG |
| SLC41A2-rev | GGCCAGTGATGCACAGAAG |
| SLC41A2-probe | CATGCTGTGCCTCCAATAAAGCAGA |
|  |  |
| FAK-1-fw | ACCTGCCGAGAAGCTGAG |
| FAK-1-rev | CGAAGAGTGCGGGTAATGAC |
| FAK-1-probe | TATAGTCACTGTGCCTGGCTGGAATATAG |
|  |  |
| FAK-2-fw | AGGCTCAAACACAAAGCAACTG |
| FAK-2-rev | CTAACTATTCTGGACCGGGAATG |
| FAK-2-probe | TGACAGGAATCCTCCACTAGACAGAAGA |

Table S2. Characterization of FAK expression (IHC), FAK copy number log ratio, and PIK3CA mutation in 67 ovarian clear cell carcinomas.

| Sample ID | Copy number | Copy number | IHC score | PIK3CA mutation |
| --- | --- | --- | --- | --- |
|  | log ratio | estimation |  |  |
| 1 | 1.548 | 6 | 2 | WT |
| 2 | 1.2605 | 5 | 3 | WT |
| 3 | 1.1875 | 5 | 2 | WT |
| 4 | 0.858 | 4 | 3 | WT |
| 5 | 0.7755 | 3 | 3 | WT |
| 6 | 0.7685 | 3 | 2 | WT |
| 7 | 0.76825 | 3 | 3 | WT |
| 8 | 0.6571 | 3 | 2 | E545K |
| 9 | 0.6225 | 3 | 3 | WT |
| 10 | 0.5805 | 3 | 3 | H1047R |
| 11 | 0.58 | 3 | 2 | WT |
| 12 | 0.51 | 3 | 3 | WT |
| 13 | 0.462 | 3 | 2 | E542Q |
| 14 | 0.4205 | 3 | 3 | WT |
| 15 | 0.3985 | 3 | 2 | WT |
| 16 | 0.3805 | 3 | 3 | WT |
| 17 | 0.377 | 3 | 3 | E545K |
| 18 | 0.372 | 3 | 2 | WT |
| 19 | 0.36075 | 3 | 2 | WT |
| 20 | 0.3395 | 3 | 1 | WT |
| 21 | 0.3265 | 3 | 1 | WT |
| 22 | 0.32025 | 3 | 1 | WT |
| 23 | 0.3125 | 2 | 2 | WT |
| 24 | 0.278 | 2 | 2 | H1047R |
| 25 | 0.2735 | 2 | 1 | H1047R |
| 26 | 0.23 | 2 | 1 | WT |
| 27 | 0.1925 | 2 | 2 | H1047R |
| 28 | 0.18 | 2 | 3 | WT |
| 29 | 0.16 | 2 | 1 | H1047R |
| 30 | 0.1455 | 2 | 2 | WT |
| 31 | 0.1435 | 2 | 1 | WT |
| 32 | 0.123 | 2 | 1 | E542Q |
| 33 | 0.0785 | 2 | 2 | WT |
| 34 | 0.026 | 2 | 1 | WT |
| 35 | -0.029 | 2 | 1 | WT |
| 36 | -0.06 | 2 | 1 | WT |
| 37 | -0.0645 | 2 | 1 | WT |
| 38 | -0.079 | 2 | 1 | WT |
| 39 | -0.1 | 2 | 0 | WT |
| 40 | -0.1125 | 2 | 2 | E545K |
| 41 | -0.132 | 2 | 1 | WT |
| 42 | -0.155 | 2 | 3 | WT |
| 43 | -0.209 | 2 | 1 | WT |
| 44 | -0.2345 | 2 | 1 | WT |
| 45 | -0.2475 | 2 | 1 | WT |
| 46 | -0.265 | 2 | 2 | WT |
| 47 | -0.269 | 2 | 1 | WT |
| 48 | -0.272 | 2 | 1 | H1047R |
| 49 | -0.334 | 2 | 0 | E542K |
| 50 | -0.3425 | 2 | 1 | ND |
| 51 | -0.352 | 2 | 3 | WT |
| 52 | -0.392 | 2 | 1 | WT |
| 53 | -0.399 | 2 | 2 | WT |
| 54 | -0.4 | 2 | 2 | WT |
| 55 | -0.41 | 2 | 1 | WT |
| 56 | -0.417 | 1 | 2 | E542K |
| 57 | -0.4315 | 1 | 2 | WT |
| 58 | -0.4545 | 1 | 1 | WT |
| 59 | -0.4825 | 1 | 2 | WT |
| 60 | -0.5145 | 1 | 2 | WT |
| 61 | -0.5225 | 1 | 1 | WT |
| 62 | -0.525 | 1 | 1 | WT |
| 63 | -0.5375 | 1 | 3 | H1047R |
| 64 | -0.5765 | 1 | 1 | WT |
| 65 | -0.625 | 2 | 1 | WT |
| 66 | -0.682 | 1 | 0 | WT |
| 67 | -0.86 | 1 | 1 | ND |

Table S3. FAK copy number log ratio and estimated FAK copy number in ovarian cancer cell lines.

| Cell Name | Histological Subtype | FAK copy number log ratio | | Pearson correlation coefficient, r | Estimated mean copy number |
| --- | --- | --- | --- | --- | --- |
|  |  | CCLE^¶^. | qPCR |  | qPCR |
| SNU8 | adenocarcinoma | -0.3 | -0.89 | 0.95 | 1 |
| OVMANA | clear cell carcinoma | -0.25 | -0.59 |  | 1 |
| RMGI | clear cell carcinoma | -0.05 | -0.26 |  | 2 |
| TOV21G | clear cell carcinoma | -0.04 | -0.01 |  | 2 |
| A2780 | adenocarcinoma | -0.04 | -0.07 |  | 2 |
| SKOV3 | adenocarcinoma | 0.05 | 0.47 |  | 3 |
| OVTOKO | clear cell carcinoma | 0.2 | 0.24 |  | 2 |
| OVSAHO | adenocarcinoma | 0.47 | 0.87 |  | 4 |
| ES2 | clear cell carcinoma | 0.5 | 0.6 |  | 3 |
| OVISE | clear cell carcinoma | 0.74 | 0.49 |  | 3 |
| SNU119 | adenocarcinoma | 1.88 | 2.42 |  | 11 |

¶. These data were downloaded from CCLE database

Table S4. Mutation status of genes in PI3K/AKT signaling pathways in ovarian cancer cell lines.

| Cell Name | Hist Subtype1 | PTK2_CN | PIK3CA | PTEN | MTOR | PDK1 | KRAS |
| --- | --- | --- | --- | --- | --- | --- | --- |
| SNU8 | adenocarcinoma | -0.3023 | WT | WT | WT | WT | p.G12D |
| OVMANA | clear_cell_carcinoma | -0.2494 | p.E545V | WT | WT | WT | WT |
| RMGI | clear_cell_carcinoma | -0.045 | WT | WT | WT | WT | WT |
| TOV21G | clear_cell_carcinoma | -0.0394 | p.H1047Y | p.L265fs, p.R142fs | WT | WT | p.G13C |
| A2780 | adenocarcinoma | -0.0388 | p.E365K | p.KGR128del | WT | WT | WT |
| SKOV3 | adenocarcinoma | 0.0541 | p.H1047R | WT | WT | WT | WT |
| OVTOKO | clear_cell_carcinoma | 0.1956 | WT | WT | WT | WT | WT |
| OVSAHO | adenocarcinoma | 0.4694 | WT | WT | WT | WT | WT |
| ES2 | clear_cell_carcinoma | 0.4963 | WT | WT | WT | WT | WT |
| OVISE | clear_cell_carcinoma | 0.7357 | WT | WT | WT | WT | WT |
| SNU119 | adenocarcinoma | 1.884 | WT | WT | WT | WT | WT |

¶. These data were downloaded from CCLE database.

**Supporting Information Legends**

**Figure S1. FAK copy number and protein levels in ovarian cancer cell lines.** A, FAK copy number log ratio (tumor vs. normal) were determined with quantitative real-time PCR. High level copy number gain was seen in the SNU-119 ovarian adenocarcinoma cell line and low level copy number gains in the SKOV3, OVSAHO, ES2, and OVISE cell lines. Copy number loss, probably 1 copy, was seen in the cell lines SNU-8 and OVMANA. Our copy number estimates were highly correlated with those downloaded from Cancer Cell Line Encyclopedia (CCLE) database (>95%, Supplemental Table 3). B, Western blottings were done to examine basal expression levels of FAK and PYK2 in 9 ovarian cancer cell lines. PYK2 was highly expressed in the OVTOKO, OVMANA, and OVISE cell lines. C, Each band intensity of FAK immunoblot result (B) was quantified and normalized with GAPDH. Normalized FAK levels (x-axis) were correlated with FAK copy number log ratios (Pearson correlation coefficient r = 0.737).

**Figure S2. Inhibition of FAK phosphorylation by FAK inhibitors in ovarian cancer cell lines.** SNU-8 (A) and TOV21G (B) cells were incubated for 24 hr in the presence of FAK inhibitors (PF271 or PF228) at the indicated concentrations (0.01 – 10 µM). FAK, PYK2, phosphorylated FAK (p-FAK, Y397) and PYK2 (p-PYK2, Y402) protein levels were determined by Western blot. GAPDH was served as a loading control. A vehicle control was performed, containing just dimethyl sulfoxide (DMSO).

**Figure S3. Synergistic effect of ABT-737 and PF271 on inducing cell death.** RMGI (A) and TOV21G (B) cells were exposed to decreasing doses of ABT-737 (A: 1, 0.5, 0.25, and 0.13 µM), PF271(P: 5, 2.5, 1.25, and 0.63 µM), or combinations (A+P) of the two agents at a fixed (1:5) ratio. After a 24-hr exposure, cytotoxicity (%) was determined by measuring the activity of released lactate dehydrogenase (LDH) in culture media using Cytotoxicity Detection Kit. Data represent mean with standard deviation (n=3).

.
